# A case of morning glory syndrome associated with persistent hyperplastic primary vitreous and Peters’ anomaly

**DOI:** 10.3205/oc000053

**Published:** 2017-01-17

**Authors:** Isil Sayman Muslubas, Mumin Hocaoglu, Serra Arf, Murat Karacorlu

**Affiliations:** 1 Istanbul Retina Institute, Istanbul, Turkey

**Keywords:** doppler ultrasonography, morning glory syndrome, persistent hyperplastic primary vitreous, Peters’ anomaly

## Abstract

We report a case of morning glory syndrome (MGS) associated with persistent hyperplastic primary vitreous (PHPV) and Peters’ anomaly. A 2-day-old girl, born at term with a birth weight of 3,350 g was diagnosed with Peters’ anomaly, cataract, microphthalmia, PHPV, and MGS. A right limbal lensectomy and vitrectomy with stalk cauterization was performed 8 days later. No early postoperative complication occurred, the family was discharged with advice on medication, and follow-up examination was scheduled. The case report reveals the coexistence of PHPV, Peters’ anomaly, and MGS, which may suggest a genetic link.

## Introduction

In 1970, Kindler first used the term morning glory syndrome (MGS) to describe a congenital excavated optic disc malformation characterized by a central glial tuft, a radiating pattern of retinal blood vessels, and an enlarged optic disc with peripapillary pigmentation. Most reported cases have been unilateral, sporadic, and in females. It has been suggested that the pathogenesis of MGS is related to poor development of the lamina cribrosa and posterior sclera [1].

Persistent hyperplastic primary vitreous (PHPV) is a congenital developmental disorder of the eye which is the result of a failure of the primary vitreous and the hyaloid vasculature to regress in the time of development of the secondary vitreous. [2] PHPV is usually unilateral and sporadic and characterized by the presence of white vascularized retrolental tissue, microphthalmia, elongation of the ciliary processes, and varying degrees of lenticular opacification [2].

Peters’ anomaly was described by Peters in 1906 in patients with a shallow anterior chamber, synechia between the iris and cornea, central corneal leukoma, and defect in the Descemet membrane. More recently, this anomaly has been subdivided into 3 groups as Peters’ anomaly types I and II and Peters’ plus syndrome. Most cases are sporadic and attributable to specific gene mutations [3].

Although MGS, PHPV, and Peters’ anomaly have been seen as isolated ocular diseases, the association of MGS and PHPV [4], Peters’ anomaly and PHPV [5], and MGS with many other abnormalities (including PHPV, Peters’ anomaly, congenital cataract, total retinal detachment, and a falciform retinal fold) have been reported [6]. 

In the present study, a case of MGS associated with persistent hyperplastic primary vitreous and Peters’ anomaly was identified, and the clinical presentation and treatment outcome are reported.

## Case description

**Figure 1:**
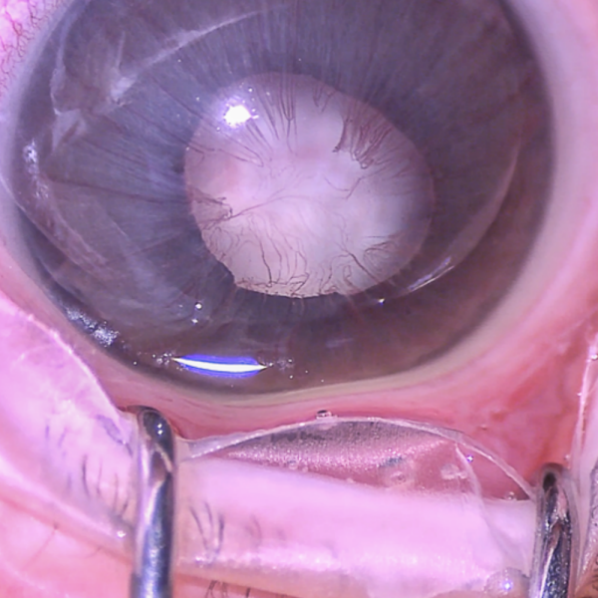
Anterior segment photograph of the patient’s right eye

A 2-day-old girl, born at term with a birth weight of 3,350 g, had a white pupil in the right eye present at birth. At the first examination, central corneal opacity, a defect in the Descement membrane, and a poorly formed anterior chamber were observed in the right eye (Figure 1 (Fig. 1)).

The intraocular pressure was 13 mmHg in the right eye and 14 mmHg in the left. The corneal diameter was 9 mm x 9 mm in the right eye and 10 mm x 10 mm in the left. The details of the fundus could not be seen in the right eye. No abnormality was detected in the left eye. 

**Figure 2:**
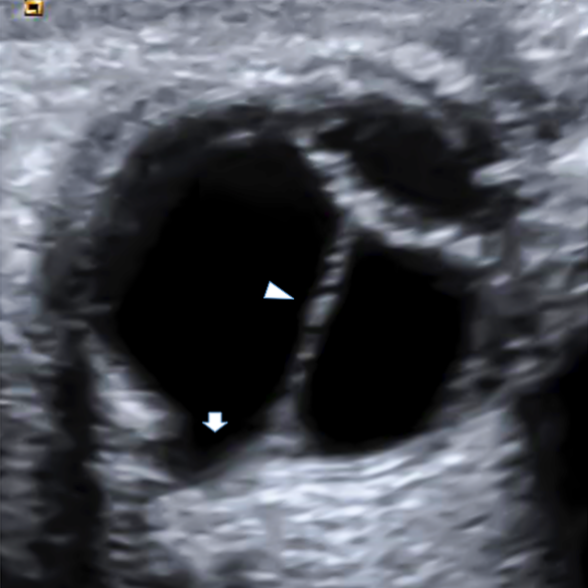
Cataract, a retrolental vascularized mass extending from the optic disc to the posterior lens capsule, and depression and enlargement of the optic disc in the right eye by Doppler ultrasonography

Doppler ultrasonography imaging showed a shortened axial length, cataract, a retrolental vascularized mass extending from the optic disc to the posterior lens capsule, and depression and enlargement of the optic disc and surrounding tissue toward the posterior pole in the right eye (Figure 2 (Fig. 2)). No pathological findings were seen in the left eye. 

**Figure 3:**
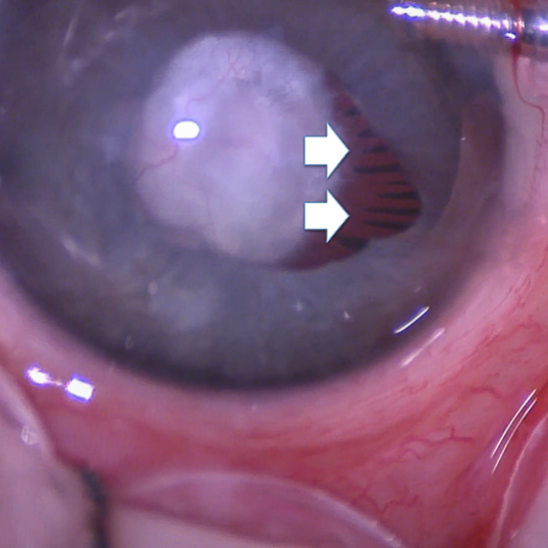
The elongation of ciliary processes was demonstrated.

**Figure 4:**
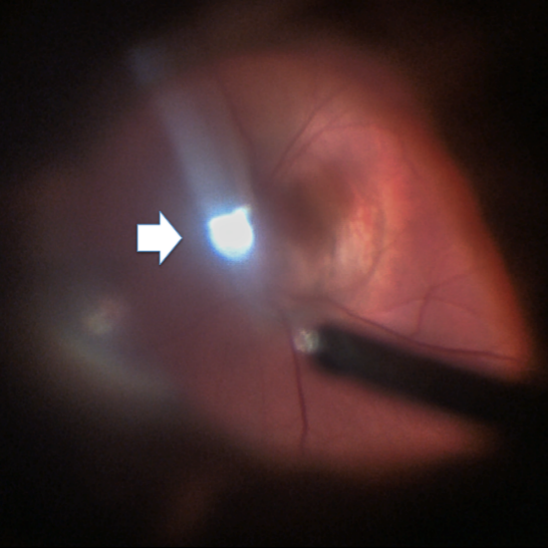
The posterior segment photograph of the patient showed depression and enlargement of optic disc.

The patient was diagnosed with Peters anomaly, cataract, microphthalmia, PHPV, and MGS. No associated systemic disorder was detected by the pediatrician. The patient’s family was informed of the diagnosis. The natural history and the success rates, risks and complications related to the surgery were explained in detail. A right limbal lensectomy and vitrectomy with stalk cauterization was performed 8 days later. The elongation of ciliary processes and enlargement of the optic disc were also observed intraoperatively (Figure 3 (Fig. 3), Figure 4 (Fig. 4)). 

No early postoperative complication occurred, the family was discharged with advice on medication, and follow-up examination was scheduled.

## Discussion

Morning glory syndrome and its many associated ocular anomalies have been reported previously [1]. In a histopathologic study, a case of MGS with many other anomalies including PHPV, Peters anomaly, congenital cataract, total retinal detachment, and a falciform retinal fold was reported and it was suggested that those associations could be coincidental or a manifestation of a neural-crest derived mesoectodermal disturbance [6]. Cennamo et al. [7] reported a case of MGS associated with PHPV and lens colobomas and speculated that those diseases were expressions of a single pathogenic process. Recently, Fei et al. [4] reported that a significant number of MGS cases was associated with PHPV and suggested a possible genetic link between the two diseases. Matsubara et al. [5] described two cases with Peters anomaly and PHPV, and migratory disorders of neural-crest cells from 4 to 7 weeks of gestation were thought to be responsible for those ocular anomalies. 

In the present study, it was thought that MGS with PHPV and Peters anomaly might have a genetic basis. The PAX6 gene is involved in ocular morphogenesis and is expressed in the developing central nervous system and various ocular tissues during development [8]. PAX6 mutations have been detected in many ocular anomalies including Peters anomaly, congenital cataract, MGS, and PHPV [8]. The coincidence of MGS with PHPV and Peters anomaly may be related to PAX6 mutations, but the assumption needs further study including genetic analysis. 

Ultrasonography examination is essential for individuals who are uncooperative or unsuitable for fundus examination owing to media opacity [9]. As in the present study, Doppler ultrasonography is needed to establish the accurate diagnosis by demonstrating MGS with PHPV. 

Visual deprivation secondary to MGS, PHPV, and Peters anomaly can result in sensory deprivation amblyopia, and management of early amblyopia is important for optimizing visual acuity. Without treatment, PHPV can also cause recurrent intraocular hemorrhage, secondary glaucoma, and then a need for enucleation and lensectomy with vitrectomy as the preferred surgical approach [10]. To eliminate the central corneal opacity of Peters anomaly, penetrating keratoplasty is one of the treatment methods, but the decision for surgery is not easy because of the higher rate of graft failure and difficulties of postoperative care in children. Medical treatment is attempted when the media opacity is not too dense [4]. In this case, lensectomy and vitrectomy with stalk cauterization were performed and amblyopia treatment for the media opacity and MGS was scheduled. 

In conclusion, the case report reveals the coexistence of PHPV, Peters anomaly, and MGS, which may suggest a genetic link. 

## Notes

### Competing interests

The authors declare that they have no competing interests. 

No payment or services have been received from a third party for any aspect of the submitted work, including design, data collection, analysis, or interpretation of the data, writing of the report, or in the decision to submit the article for publication.
